# Inference of Lineage-Resolved Cell Identities in Uncompressed *C. elegans* Embryos

**DOI:** 10.17912/micropub.biology.002192

**Published:** 2026-05-23

**Authors:** Miles P Tran, Neil Peinado, Zhongying Zhao, Pavak K Shah

**Affiliations:** 1 Bioinformatics Interdepartmental Program, University of California Los Angeles; 2 Molecular Biology Interdepartmental Program, University of California Los Angeles; 3 Department of Biology, Hong Kong Baptist University; 4 Molecular, Cell and Developmental Biology, University of California Los Angeles

## Abstract

Lineage-based cell identification in *C. elegans* typically requires intensive live imaging and tracking. Recent methods attempt to automate cell identification on the basis of spatiotemporal atlases. We present EmbAlign, an automated 3D registration framework that determines lineage identities from single embryo snapshots. EmbAlign retrieves reference templates from a spatiotemporal atlas and refines assignments using an iterative Sinkhorn alignment procedure, robustly handling positional variability and arbitrary orientations in uncompressed embryos. EmbAlign achieves 96.9% accuracy up to the 190-cell stage and includes a diagnostic layer for continuous scoring (AUPRC = 0.546), converting raw spatial data into lineage aware datasets.

**Figure 1. EmbAlign enables fully automated cell labeling in C. elegans embryo snapshots:** A. Overview of the EmbAlign framework. Input Centroids: Raw 3D unoriented input centroids (top) and candidate reference templates retrieved from the spatiotemporal atlas (bottom). Coarse Alignment: Superposition of observed centroids with candidate reference template (top). A discrete rotational sweep around the principal component axis and the resulting cost landscape (bottom). Sinkhorn Refinement: Iterative Sinkhorn refinement mapping the spatial correspondence between observed centroids and the candidate reference (top) along with the resulting cost landscape (bottom). Label Assignment: The final discrete lineage identity assignments (top) and the resulting distribution of per-cell confidence scores (bottom) generated by the diagnostic layer. B. Cross-validated, per-frame alignment accuracy (N=11, mean = 96.9%) aligned to a canonical time axis. Rolling mean frame accuracy (solid black) with bootstrapped 95% confidence intervals (shaded). C. Per-frame alignment accuracy for two independently generated light sheet microscopy datasets. D. Distribution of assignment accuracies across all training embryos for the 30 cell types with the lowest overall mean accuracy. E. Cross-validated AUPRC curves (mean AUC = 0.546). Mean interpolated precision (solid blue) and bootstrapped 95% confidence intervals (shaded). Naive baseline (0.05, dashed). F. Normalized confusion matrix. Values indicate the proportion of alignments correctly classified within each true class. G. Side-by-side comparison of ground truth label assignment accuracy (left) and the predicted confidence score (right).

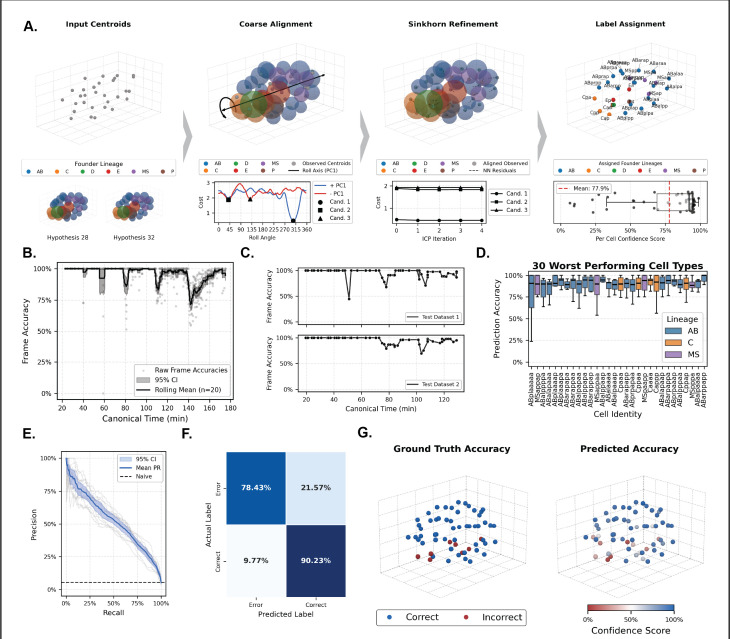



## Description

During the mapping of the *C. elegans* embryonic lineage (Sulston et al., 1983) , the spatial stereotypy of the embryo was noted. More recent automated tracking-based lineage tracing (Bao et al., 2006; Santella et al., 2010, 2014) enabled quantitative assessment of the consistency of cell positioning within the embryo (Li et al., 2019; Moore et al., 2013; Schnabel et al., 2006) , a feature that has led to recent advances (Haus et al., 2025; Ntemos et al., 2025) in using spatial atlases to automate cell identity determination from static snapshots of *C. elegans* embryos using timelapse recordings of embryos imaged under gentle compression. Compression has been extensively used in prior live imaging experiments (Sulston et al., 1983) as it forces embryos into stereotypical orientations and limits their axial extent, allowing for 3D coverage with fewer focal planes in confocal imaging (Bao & Murray, 2011) . While current approaches achieve high accuracy in the inference of lineage identity from static snapshots of cell position, both of these tools were trained using compressed data. Because this mechanical compression physically flattens the Z-axis, it alters the relative spatial topology of nuclei. Consequently, tools trained on these compressed datasets may struggle to map the 3D geometry of uncompressed embryos, which are free to rotate in space. We built EmbAlign to fill this gap by enabling robust lineage identity determination using cell position in uncompressed embryos, such as would be generated by many smFISH (Parker et al., 2021) and immunofluorescence (Duerr, 2006; Parker et al., 2021) sample preparation approaches, and by snapshots from uncompressed live imaging, for example by lightsheet microscopy (Wu et al., 2011) . In the case of assays like smFISH or immunofluorescence, embryos can be fixed in suspension, preserving their native 3D architecture while inherently precluding any live imaging. EmbAlign provides the geometric inference to map molecular profiles to lineage identities in these preparations.

EmbAlign automates the transformation of 3D nuclei centroids from uncompressed C. elegans embryos onto their canonical lineage identities ( **Fig. 1A** ). While users can generate these coordinates using various 3D segmentation strategies, ranging from classical Laplacian of Gaussian blob detection (Kong et al., 2013) to deep-learning models like Cellpose (Stringer et al., 2021) , robust detection is critical. Before executing EmbAlign, we recommend visually verifying segmented centroids against raw fluorescence images as a standard practice. Once these validated 3D centroids are obtained, they are centered and scaled to standardize physical size variations while preserving relative spatial topology. Using the total observed cell count, the algorithm selects from reference templates derived from empirically observed lineage identity configurations. To resolve arbitrary orientations, the algorithm aligns the observed centroids' primary principal component (±PC1) axes to the reference and executes a discrete rotational sweep to identify the top k unique angular orientations. Each initialization is then refined via an iterative Sinkhorn alignment procedure. This stage utilizes entropically regularized optimal transport to compute a soft correspondence probability matrix and iteratively updates the best fit rigid transformation. Following refinement, EmbAlign resolves a final, discrete mapping of identities to centroids by minimizing the Sinkhorn-weighted Mahalanobis distance, enforcing the one-to-one correspondence required by the embryo's invariant body plan. Finally, a Random Forest diagnostic layer evaluates the assignment, utilizing geometric and biological features to output a continuous confidence score for every prediction.&nbsp;

EmbAlign's performance was evaluated using a Leave-One-Out Cross-Validation (LOOCV) strategy across a dataset of uncompressed C. elegans embryos from two different labs acquired using distinct imaging modalities (embryo n = 11, frame n = 1,215), where ground truth cell identities were established using the AceTree (Boyle et al., 2006; Katzman et al., 2018) /StarryNite (Bao et al., 2006; Santella et al., 2010, 2014) lineage tracking pipeline. Position data for uncompressed embryos were sourced from previously published lightsheet microscopy lineage reconstructions. The algorithm achieved a high average frame accuracy of 96.6% from the 6-cell up to the 190-cell stage ( **Fig. 1B** ). Despite this overall robustness, performance exhibited predictable transient declines corresponding to waves of synchronous cell division. Nuclei captured within or immediately adjacent to these division windows are inherently more difficult to classify, as rapid physical displacement during cytokinesis briefly deforms the expected spatial topology and maximizes the distance of these cells from their canonical atlas positions. Finding an exact reference template match is inherently challenging during these rapid waves of cell division. Remarkably, EmbAlign maintains a 86.6% assignment accuracy even when aligning against an imperfect reference template (n = 270), compared to a near-perfect 99.4% accuracy when at least one exact reference template match is available (n = 945). This suggests the EmbAlign framework successfully buffers against missing reference templates, and increasing training dataset size to more robustly cover gaps in the current reference atlas should further improve overall performance.

To verify that the pipeline does not overfit to the spatial properties of the training data, we evaluated EmbAlign on two fully independent, out-of-sample (OOS) datasets acquired via single view selective plane illumination microscopy (ASI diSPIM operated in single view acquisition mode). These OOS embryos were annotated with ground truth labels using AceTree and StarryNite pipeline up to the 100 cell stage. The algorithm successfully mapped these embryos, maintaining comparable high-fidelity frame accuracies (96.3% and 96.1%) without any dataset-specific recalibration ( **Fig. 1C** ). This OOS performance demonstrates robust generalizability, confirming that EmbAlign effectively captures the invariant spatial dynamics of *C. elegans* embryogenesis rather than batch-specific imaging artifacts.&nbsp;

To investigate cell type specific drops in performance, we examined the 30 lowest-performing cell types. We observed that these challenging types primarily consist of later-stage cells that emerge toward the end of our atlas window and correspond to a wave of cell divisions. Additionally, we correlated our global cell-type accuracies with empirical positional variance measurements from a prior study (Guan et al., 2025; Li et al., 2019) in uncompressed embryos. We found a small but significant negative correlation (Spearman' s = -0.11, p = 0.03), suggesting the natural spatial variability of these cells is likely a minor contributing factor to EmbAlign's difficulty in resolving their identities.&nbsp;&nbsp;

To enable reliable application in experimental settings lacking ground truth, we integrated a decoupled Random Forest diagnostic layer that provides a single cell assessment of alignment quality in real-time. Because the pipeline maintains a high baseline accuracy, the diagnostic task is imbalanced, requiring the classifier to identify rare misassignment events within a vast majority of correct labels. To rigorously evaluate performance under those conditions, we compared the precision (a measure of the false positive rate) and recall (a measure of the false negative rate) of the model. The diagnostic layer achieved an AUPRC of 0.525—a greater than 10-fold improvement over the naive baseline of 0.05 ( **Fig. 1E** )—and&nbsp; successfully identified 78.5% of true assignment errors and 90.2% of true assignment successes ( **Fig. 1F** ), demonstrating a robust ability to capture specific geometric and biological features of assignment failures.

To make these diagnostics accessible to the end user, we developed an interactive HTML alignment report that packages the pipeline's outputs into a comprehensive dashboard. Along with a frame level confidence estimate, the tool projects the aligned embryo onto an empirical population growth curve to estimate its canonical time, a feature that allows users to flag datasets that are likely to have been captured during transient, error-prone division windows. The dashboard also summarizes the entire search landscape, enabling users to compare alternative alignments across multiple local minima and track their respective optimization convergence traces. Finally, predicted labels and alignment confidence scores are mapped directly onto the embryo's 3D spatial topology, resulting in interactive 3D spatial label assignment and confidence plots that facilitate rapid verification of alignment quality ( **Fig. 1A, 1G** ).&nbsp;&nbsp;

While EmbAlign provides a robust framework for identity inference in uncompressed embryos, its current implementation has specific boundaries defined by training data availability and input data quality. The pipeline is validated up to the 190-cell stage: a limit dictated by the increased difficulty of generating ground truth training data for uncompressed embryos using compression-optimized tracking tools, rather than an inherent algorithmic constraint. Furthermore, because the algorithm relies on a strict one-to-one correspondence between observed nuclei and those in the candidate atlas template, EmbAlign is very sensitive to detection errors, requiring careful validation of cell detection in test data prior to alignment. However, the framework's reasonable performance on imperfect reference templates suggests that missing or extra nuclei primarily trigger localized mapping failures rather than catastrophic global misalignment. This behavior underscores the critical utility of the diagnostic layer and interactive alignment report, which projects alignment confidence scores spatially to help users visually flag and isolate these localized, artifact driven misassignments.

In summary, EmbAlign provides a generalizable solution for automated cell identity inference in static 3D snapshots of uncompressed *C. elegans* embryos. By aligning 3D nuclei centroid coordinates to live-imaging derived spatiotemporal atlases of early *C. elegans* development, EmbAlign achieves >96% alignment accuracy up to the 190 cell stage that generalizes across independently generated datasets. Additionally, we observe that performance lapses correspond to transient waves of synchronous cell division, where natural positional variance temporarily disrupts spatial stereotypy. Supported by a predictive diagnostic classifier and interactive alignment reports, EmbAlign provides a complementary tool for transforming raw spatial coordinates into lineage-aware datasets.

## Methods

**Spatiotemporal Atlas Construction** &nbsp;

Embryos were first aligned to a canonical time axis via dynamic time warping ( *Dynamic Programming Algorithm Optimization for Spoken Word Recognition* , n.d.) . A continuous spatial reference was constructed by fitting independent 1D Gaussian Process regressors (RBF and White kernels) to the spatiotemporal trajectories of each cell type's 3D coordinates. Total positional variance at time t was calculated by aggregating the GP regression uncertainty with empirical cell-specific variance.&nbsp;

To define the database of biologically valid cell combinations (“slices”), we utilized strictly empirically observed states. Slices were extracted directly from the training frames by aggregating valid cell label combinations grouped by frame cell count (N). Finally, discrete slices were inflated into function 3D reference frames. We calculated the overlapping temporal existence window, the canonical time window within which all cells in a given slice simultaneously exist, for each observed slice and queried the continuous GP atlas at the window's midpoint (t _med_ ) to retrieve the expected 3D coordinates and covariance matrices required for alignment. To provide a temporal baseline for downstream diagnostic validation, an empirical growth curve was also constructed by calculating the mean and standard deviation of total cell counts across canonical time bins in the training data.

**EmbAlign 3D Alignment and Label Transfer**

Observed 3D nuclei centroids were first centered and scaled by their median pairwise distance. For each candidate atlas slice matching the observed cell count N, we aligned both the positive and negative primary principal component (±PC1) axes of observation to the reference PC1 axis to account for PCA sign ambiguity. We then rotated the observation around the PC1 axis in discrete angular steps, scoring each orientation based on the Sinkhorn-weighted squared Euclidean distance between observed and reference centroids. To escape geometric symmetry traps, we identified the top k unique angular valleys (separated by ≥30°) to seed the refinement phase.&nbsp;

Each of the k initializations entered an Iterative Closest Point Refinement (Bergström & Edlund, 2014) (ICP)-like soft refinement loop. To more robustly handle biological noise, we replaced the standard hard-assignment matching of the ICP algorithm with optimal transport. We computed a soft correspondence probability matrix using Sinkhorn entropic regularization. The rigid transformation (rotation and translation) was then iteratively updated using a weighted Kabsch Algorithm (Kabsch, 1976) , where the influence of each cell pairing was governed by its Sinkhorn probability.&nbsp;

Alignments were scored by calculating the Mahalanobis distance between the aligned observations and the atlas 3D Gaussian distributions. The slice and orientation minimizing this global cost were selected as the winner. Final discrete cell identities were assigned by executing a linear sum assignment algorithm (Kuhn, 1955) directly on the winning mahalanobis distance matrix.

**Confidence Scoring**

A Random Forest (Breiman, 2001) classifier (n_estimators = 200, max_depth = 10) was trained to predict cell-level assignment correctness using geometric and biological alignment features. Input variables included Sinkhorn assignment entropy, Mahalanobis distance, frame cell count, frame inferred time, and a normalized division delta (t _med_ -tbirth)/(t _division_ -tbirth) representing cell life progress. The model outputs continuous cell-level probabilities, which are also averaged to generate an aggregate frame level confidence score.&nbsp;

Pipeline accuracy and diagnostic accuracy were evaluated using a Leave-One-Out cross-validation strategy. For each fold, the continuous spatial GP atlas, slice atlas, and diagnostic classifier, were fitted entirely on the training embryos prior to evaluating the withheld test embryo.

**QC Report Generation**

To facilitate rapid visual assessment of alignment quality, the pipeline generates an interactive HTML diagnostic dashboard. To trace the optimization landscape, the alignment engine records the Sinkhorn-weighted sum of squared Euclidean distances at each discrete step of the coarse angular sweep. Furthermore, for the top k angular initializations selected for the refinement tournament, this same soft-weighted cost is sequentially tracked across all Sinkhorn refinement loops. These convergence traces are packaged alongside the empirical population growth curve, which plots the unannotated embryo's observed cell count and t _med_ against the 95% confidence intervals of the training population.&nbsp;

**Data Acquisition&nbsp;**

For validation, JIM113 embryos were isolated from gravid hermaphrodites and mounted on a coverslip using a diSPIM sample chamber. Images were acquired with 0.75 micron z-spacing on an ASI diSPIM (Wu et al., 2013) run in single view acquisition mode controlled by the diSPIM control plugin in micro-manager (A. Edelstein et al., 2010; A. D. Edelstein et al., 2014) . Images were cropped in ImageJ (Schneider et al., 2012) and processed using StarryNite. Lineage tracing results from StarryNite were then manually curated using AceTree.

**Data and Code Availability**

The EmbAlign source code is available as a public repository at https://github.com/shahlab-ucla/EmbAlign. This repository also contains executable scripts, configuration files, and pretrained models required to reproduce the analyses and figures presented in this study. All processed datasets used for model training and out-of-sample validation are hosted within the repository and permanently archived at https://doi.org/10.5281/zenodo.20089241. The repository also contains core usage vignettes outlining execution of the EmbAlign pipeline for lineage inference in unlabeled 3D nuclei coordinates, as well as instructions for fitting the spatiotemporal atlases on raw point cloud data.

## Data Availability

Description: v1 release of EmbAlign source code, also indexed at https://doi.org/10.5281/zenodo.20089241 provided under the MIT license.. Resource Type: Software. DOI: https://doi.org/10.22002/w3xfh-10x37
